# Neurosurgical Equipment Donations: A Qualitative Study

**DOI:** 10.3389/fsurg.2021.690910

**Published:** 2022-01-20

**Authors:** Dawin Sichimba, Soham Bandyopadhyay, Ana Catinca Ciuculete, Joshua Erhabor, Jay Kotecha, Abdullah Egiz, Nourou Dine Adeniran Bankole, George Higginbotham, David Ulrich Dalle, Ulrick Sidney Kanmounye

**Affiliations:** ^1^Department of Research, Association of Future African Neurosurgeons, Yaounde, Cameroon; ^2^Michael Chilufya Sata School of Medicine, Copperbelt University, Kitwe, Zambia

**Keywords:** neurosurgical, equipment, donations, donor, developing countries, UK, low-and-middle income countries (LMICs)

## Abstract

**Introduction:**

Neurosurgical equipment donation from high-income countries (HICs) to low-and-middle income countries (LMICs) exists. However, there is currently no published literature on whether there is a need for neurosurgical equipment donations or how to design equipment donation programmes that meet the needs of LMIC neurosurgeons. The primary aims of this study were to explore: (1) the need for the donation of neurosurgical equipment from the UK and Ireland to LMICs within the African continent, and (2) the ways through which neurosurgical equipment donations could meet the needs of LMIC neurosurgeons.

**Methods:**

This was a qualitative study using semi-structured, one-on-one, audio-recorded interviews. Purposive sampling was used to recruit and interview consultants or attending neurosurgeons from Ireland, the UK and LMICs in Africa in a continuous process until data saturation. Interviews were conducted by members of the Association of Future African Neurosurgeons during March 2021. Qualitative analysis used a thematic approach using open and axial coding.

**Results:**

Five HIC and 3 LMIC neurosurgeons were interviewed. Five overarching themes were identified: (1) inequality of access to neurosurgical equipment, (2) identifying specific neurosurgical equipment needs, (3) importance of organisations, (4) partnerships between LMIC and HIC centres, and (5) donations are insufficient in isolation.

**Conclusion:**

There is a need for greater access to neurosurgical equipment in LMICs. It is unclear if neurosurgical equipment donations are the optimal solution to this issue. Other solutions that are not linked to dependency need to be explored and executed. Collaborative relationships between LMICs and HICs better ensures that neurosurgical equipment donations meet the needs of the recipients. These relationships may be best created within an organisation framework that has the logistical capabilities of coordinating international equipment donation and providing a quality control measure.

## Introduction

Of the 13.8 million individuals requiring neurosurgical care each year, more than 80% are located in low-and-middle-income countries (LMICs) (1). Despite their disproportionate neurosurgical needs, LMICs have significantly fewer human resources, funding and infrastructure to meet them compared to high-income countries (HICs) (2, 3). The World Federation of Neurosurgical Societies' Global Neurosurgery Committee (WFNS GNC) recommends countries should have at least 1 neurosurgeon per 200,000 inhabitants, universal health coverage for all neurosurgical emergencies, and universal 2-h access to a facility that provides basic macroneurosurgery (4). While there have been considerable improvements in neurosurgical workforce and universal health coverage for neurosurgical emergencies, most LMICs face significant infrastructural challenges (5, 6). The WFNS GNC mapped global access to neurosurgical infrastructure using a three-tier categorisation and geographic information systems: Level 1 facilities have the resources to provide basic macroneurosurgery; especially emergency neurotrauma care; Level 2 facilities provide basic microneurosurgery in addition to macroneurosurgery; and Level 3 facilities provide advanced microneurosurgery in addition to basic microneurosurgery and macroneurosurgery (7). This mapping project revealed that most LMICs do not have enough neurosurgical facilities to cover their entire population (7). Similarly, LMIC centres have limited access to neuro-intensive care units, microsurgery equipment, and intraoperative guidance, resulting in LMICs being less likely to provide Level 2 and 3 care (8). Karekezi et al. evaluated the impact of African neurosurgeons whose training had been sponsored by the WFNS and found that restricted access to neurosurgical equipment limited the neurosurgeons' service delivery in their home countries (9).

Mindful of these challenges, strategies of neurosurgical equipment donation from HICs to LMICs have been implemented (10, 11). These initiatives were largely created to tackle the health inequity mentioned above and advance the agenda for social justice for LMIC neurosurgical patients. Social justice in the realms of healthcare can be thought broadly as advocating for individuals' ability to receive timely and adequate treatment independent of their background characteristics, including geographical location (12). Despite neurosurgical equipment donation programmes being set up with the best of intentions, there exists no literature establishing whether neurosurgery equipment donations programmes were needed. The existing literature on neurosurgical equipment donations are sparse, quantitative, and from the lens of HIC authors (10, 11). They solely focus on the impact of a neurosurgery equipment donation initiative. Whilst quantitative methods can be used to assess the impact of a neurosurgical equipment donation, they impose a predetermined metric through which to measure impact based on predetermined beliefs. Therefore, a quantitative methodological approach is too rigid to deeply explore the need for neurosurgical equipment donations or design effective equipment donation programmes that meet the needs of LMIC neurosurgeons (13).

Given the relation of the above to social justice, it is important that the research method used acts as a vehicle through which social justice can be enacted. Qualitative research shares several elements that are in keeping with the pursuit of social justice (14, 15). Primarily, there is a recognition that context is critical (16–18). Secondly, great emphasis is placed on creating a reciprocal relationship between the researchers and research participants, and reflecting on the effect this relationship might have had on the results (17, 19). By enabling this focus on equity, access, participation and harmony, qualitative research allows researchers to assist the people participating in the study in a socially just manner (15, 20). For the purpose of this study, qualitative research design was utilised through the contextualised study of individuals through interviews. Interviews are a means through which both the participants' words and the meaning behind those words can be captured (21). This enabled for more detailed answers, freedom in discussion, and participants to expand on their thoughts and experiences in their own words (22). This in turn allowed for topic areas to be brought up by participants that were not directly asked about by the interviewer. Given the time- and resource-intensive nature of qualitative research, it is important to state that the study design lends itself to small sample sizes. The rigour of qualitative research depends on reflexivity rather than recruiting a predetermined number of participants (23), and recruiting more participants to increase the size of the dataset would do as much to compromise the depth of the analysis as to increase its breadth (24).

Given the researchers conducting this study were affiliated with the Association of Future African Neurosurgeons, it was determined that the focus of this study would be within the geographical areas that the researchers were located in. Therefore, the study focussed on the perspectives of HIC neurosurgeons in the United Kingdom (UK) and Ireland, as well LMIC neurosurgeons in Benin, Cameroon and Zimbabwe. The primary aims of this study were to explore (1) the need for the donation of neurosurgical equipment from the UK and Ireland to LMICs within the African continent, and (2) the ways through which neurosurgical equipment donations could meet the needs of LMIC neurosurgeons. The secondary aims of this study were to identify (1) the views of attending or consultant neurosurgeons regarding the topic of neurosurgical equipment donations, (2) the barriers to donating neurosurgical equipment, and (3) the factors that could motivate neurosurgical equipment donation.

## Methods

### Study Design

This study was designed by authors who all met and connected online via social media channels. The authors connected over their shared interest of neurosurgery and equipment donations. This was a qualitative study using semi-structured one-on-one audio-recorded interviews (25). A participatory approach was taken. Participants were actively involved in the research process and the co-creation of understandings. This study received ethical approval by the University of Oxford Medical Sciences Inter-Divisional Research Ethics Committee (Ethics Approval Reference: R74097/RE001) on 15th February 2021. Participation was voluntary and informed consent was obtained from each participant before embarking on this study.

### Participants

Unlike sampling in quantitative studies where the goal is to randomly sample a population with the intention of making inferences from that sample to the population in general, qualitative research requires purposive sampling that focuses on particular characteristics of the population of interest. As such, purposive sampling was used to recruit consultants or attending neurosurgeons from Ireland, the UK and LMICs in Africa, as defined by the World Bank criteria (26). A non-probability sampling technique was used as the aim of qualitative research is not to produce a statistically representative sample or draw statistical inference, but to have an appropriate group of individuals that reflects the diversity and breadth of the sample population (27). Indeed, an idea needed to only appear once to be deemed of value. The specific form of purposive sampling used was theoretical sampling, where recruitment and interviewing were done in a continuous process until both a suitably varied group of participants had been interviewed and when the data was deemed to have no further interpretive value, often termed data saturation (28, 29).

### Data Collection

Written informed consent was sought from all participants including for audio-recording and anonymous quotations. Demographic information was gathered from all participants including ethnicity and geographic location. Interviews were conducted using a semi-structured approach. The interviews were conducted using a topic schedule (Appendix S1). This started with specific questions but as themes arose these were followed. All interviews were conducted one-on-one with each participant over Zoom over a 4-week period in March 2021. Each interview was audio-recorded and transcribed. Audio records and anonymised transcripts were encrypted and stored in a secure location.

### Data Analysis

Qualitative data coding, management, and analysis was conducted. Identifiers were removed from transcripts to preserve anonymity. Qualitative analysis used a thematic approach using open and axial coding (28). Open coding involved deconstructing participant responses into common groupings based on shared ideas. Dominant ideas that emerged were then organised into overarching themes through axial coding. Each author independently reviewed and coded the data. Any conflicts in coding were resolved by mutual agreement. Participant data were interpreted and summarised. Codes of similar information were merged leading to a series of phenomena that appeared increasingly representative of the participants perspectives. Data gathering ceased when collecting more data was deemed to have no further interpretive value. To reduce researcher bias, we discussed and maintained an awareness of preconceptions and constantly linked the emergent themes to the interview data.

## Results

### Participants

Eight neurosurgeons participated in this study. There was a wide spectrum of experience with neurosurgical equipment donations, with some individuals having no experience and others having extensive experience. Similarly, individuals belonged to a wide spectrum of neurosurgical centres, in which some centres had never been involved in neurosurgical equipment donations, some centres had previously been involved in neurosurgical equipment donation, and some centres were currently involved in neurosurgical equipment donation. The participants represented the diversity and breadth of the sample population.

### Thematic Analysis

Analysis of the interviews resulted in five overarching themes. These are described in greater detail below and supported with verbatim quotes from study participants (Appendix S1).

#### Inequality in Access to Neurosurgical Equipment

All individuals recognised that LMICs did not have access to certain types of neurosurgical equipment, and this was a need that should be addressed.

There was diversity of thought on how best to meet this need. Some believed that donations were the best way forward. Others believed that donations created a dependency relationship, and the way forward was through the creation of an exchange system.

#### Identifying Specific Neurosurgical Equipment Needs

Neurosurgeons in HICs felt a barrier to donation was lack of knowledge about what was needed. They believed that the needs of one country did not map onto the needs of another. They agreed that neurosurgery equipment donations were only useful if the equipment donated matched the demands of the recipient centre.

This discrepancy in need was evident in the fact that some LMICs were recognised to have facilities that were comparable to those present in neurosurgical centres in HIC countries, whilst others were struggling to acquire common consumables used in neurosurgery.

Despite differences, neurosurgeons based in different LMICs shared some common needs. An equipment that was found to be wanting and in high demand was the neurosurgery operating microscope.

LMIC and HIC neurosurgeons felt the cost and the logistics of transport created a barrier to donating microscopes. These costs were considered to be balanced by some by the longevity of the donation. However, others felt there was a risk that the microscope could break down, and if there was no system in place for servicing the equipment this would lead to a waste of valuable resources.

#### Importance of Organisations

Given the complexities surrounding equipment donations, the involvement of an organisation in this process was thought to be important. Organisations were believed to be important in dealing with the logistics of collecting equipment from multiple centres in HICs, providing quality-control and infection-control of the equipment being donated, and distributing the equipment to LMIC centres.

Two organisations that were identified to be important in existing equipment donation were the World Health Organisation (WHO) and the WFNS. UK neurosurgeons believed that the Society of British Neurological Surgeons (SBNS), as a member society of the WFNS, were optimally placed to bring together the UK neurosurgical community for the purposes of equipment donation between centres within the UK and abroad. The British Medical Association (BMA) and the Royal College of Surgeons in Ireland (RCSI) were thought to be other organisations who could potentially take up this role in the UK and Ireland, respectively. However, involvement through WFNS was highly favoured by LMIC neurosurgeons.

A concern regarding organisations distributing neurosurgical equipment was the lack of transparency and auditing of where the material had been distributed to. It was theorised that being able to follow up the positive effects of an equipment donation could potentially advertise the benefits of doing so and encourage future equipment donations. It was appreciated that this may be difficult to do so for an organisation dealing with the inflow and outflow of equipment worldwide, and in the long-term having multiple organisations involved could improve the efficiency and transparency of this system. It was also thought to be important for the organisation involved in this process to ensure donating centres were not taking away equipment that would be needed for their own patients, as this could introduce significant opposition to equipment donation. A way to bypass this issue was identified to be for reusable, expensive equipment to be donated when hospitals in HICs were upgrading their equipment.

#### Partnerships Between LMIC and HIC Centres

The importance of relationships between centres was a key tenet of promoting neurosurgical equipment donations. A HIC neurosurgeon having a personal relationship with another neurosurgeon in a LMIC centre was a key motivator for donating to that centre.

These partnerships also better enabled the equipment donations to match the specific needs of the LMIC centre. Partnerships to establish needs could also be with neurosurgical societies based in different countries.

However, there were some qualms about how partnerships are largely created between people who know each other or where there are historical links between centres. This could place some centres without this network at a disadvantage to receiving equipment from HIC centres. It was thought to be critical to have an organisation act as a mediator to set up partnerships where none exist.

#### Equipment Donations Are Insufficient in Isolation

Partnerships also enabled training of local neurosurgeons to be performed. The educational side to these international partnerships was thought to be more valuable long-term as it could optimise the usability of current and possibly future neurosurgical equipment donations. There were fears that inadequate training of how to use a neurosurgical equipment could lead to harm being done to the local population or the equipment being broken.

There was a consensus that this training needed to occur in the setting of the LMIC centre, so that the equipment could be taught to be used within the resource constraints and environment of the LMIC that would be using it henceforth. The importance of training LMIC neurosurgeons was also highlighted in the fact that HIC neurosurgeons often came to LMIC countries to perform surgery and then went back home, and there was a need to have a local person trained to look after the patient post-operatively once the HIC neurosurgeon had left the LMIC.

Despite the recognition by HIC neurosurgeons of the need to train individuals within LMIC settings, there was some hesitancy about saying this and frequent unprompted clarifications that this was not related to denying opportunities for LMIC neurosurgeons in HICs or an act of charity. The time commitment required to spend a length of time training individuals in a LMIC away from family was highlighted as a barrier to equipment donation, as were local political situations that made educational endeavours challenging.

## Discussion

### Summary of Findings

There is inequality in access to neurosurgical equipment between neurosurgical centres, both within the context of LMICs and between LMICs and HICs. Neurosurgical equipment donations were believed to be one method by which to address this inequity. However, there were concerns this could lead to a dependency relationship, and an exchange programme would be preferred. Regardless of the means of providing the equipment to centres, there was a recognition of the importance of first establishing the needs of a centre. This could be done by establishing partnership between centres or through organisations. Bilateral partnerships were highlighted to be key in motivating individuals to continue donating. For this reason, it was suggested that organisations, who are better suited to cope with the logistics of equipment donations on an international scale, should also encourage the creation of these relationships and be transparent about the impact of each donation. Furthermore, partnerships would also encourage an aspect that is critically linked to successful neurosurgical equipment donations: education on how to use the equipment to best serve the needs of the local population. This education and training were thought to be best done within LMIC centres, so that individuals could be trained in how to use the equipment within the context of the local resources available.

### Implications

The need for neurosurgical equipment in LMICs has been highlighted in prior literature (10, 30, 31), and our current study suggests this need is still unmet. Unidirectional donation of equipment has traditionally been the approach to address this, and is still supported by some study participants. However, equipment donations were identified as possibly creating a possibly harmful power imbalance between donors and recipients, and the harmful effects of dependency that have been well documented in prior research (32, 33). However, it should be noted that donations are not intrinsically linked to dependency. Studies have shown that dependency is influenced by many factors, some of which do include the length and intensity of a donation period, but are linked to other factors that disincentivise local production and acquisition of skills (34, 35).

Our study also identified the potential for mis-matched donations resulting from a lack of understanding of recipient needs; a phenomenon that has been identified to be an issue in equipment donations for other subspecialties (30). One method to ameliorate this is through a collaborative approach, with reciprocal relationships between HICs and LMICs (30). The importance of bilateral partnerships has been noted previously, but so too have the difficulties in creating such partnerships. The greatest barrier identified in a survey by Davis *et al*. was the identification of potential partners (36). Participants in the current study validated this difficulty in neurosurgery, and called for an organisation to facilitate the equitable formation of partnerships. An example of such an organisation is the InterSurgeon platform, which was developed as a mechanism for promoting international neurosurgical collaboration (37).

The utility of organisational involvement goes beyond this. The WHO highlights how organisations may have the logistical capabilities to tackle some of the barriers to effective donation of neurosurgical equipment that were identified in this study (38, 39). For example, organisations may have the funding and manpower to track and monitor the impact of equipment donations, thereby providing greater transparency and accountability. However, there is a risk that an organisation tasked with this worldwide could grow too large and be less efficient than several smaller organisations (40, 41). A novel approach could be the creation of bilateral partnerships between HIC and LMIC centres within an organisation framework, which would combine the benefits of central organisations coordinating logistical efforts with the reciprocal understandings created through partnerships.

Interestingly, a survey of InterSurgeon members did not rank the physical sharing of equipment as a top priority of collaboration (37). Rather, the greatest importance was placed on the training of neurosurgeons in LMICs, which is in keeping with views expressed by participants in the current study. The importance of training and developing skills within local populations is also a key factor in preventing the deleterious effects of equipment donations (35). The hesitancy of some HIC neurosurgeons to embark on neurosurgical equipment donations due to their knowledge of the need to provide this training and the time commitment this would involve should be applauded, as this prevents the harm caused by short-term endeavours (42, 43). However, geographical displacement and time away from family do not need to be a barrier to providing the education that is critical for successful neurosurgical equipment donations. With the advancement of communication technology applications like Zoom, it is becoming increasingly easier to teach surgical skills through web-based applications without the need for physical travel (44).

It should be noted that neurosurgeons interviewed from HICs in this study were limited to the UK and Ireland only. It is possible that motivating factors and barriers to neurosurgical donations pertinent to neurosurgeons from other HICs were missed. Similarly, this study included neurosurgeons from African LMICs, and it is possible that neurosurgeons in other LMICs had differing opinions on the need for neurosurgical equipment donations. Therefore, the recommendations made from this article should be considered within the context of one of the primary aims of this study, which was to identify if there was need for the donation of neurosurgical equipment from the UK and Ireland to LMICs within the African continent. An additional limitation to this study was that qualitative analysis relies on the quality of the interview and the need for the interviewer not to introduce any biases. Therefore, the interviewers discussed and maintained an awareness of preconceptions and constantly linked the emergent themes to the interview data to minimise bias. In addition, a semi-structured approach was taken to the interview. This may have limited the participants from answering in a more diverse manner about their views on equipment donation, and instead directed them towards areas mentioned in the topic schedule. In order to minimise participants' answers being restricted in their content, it was essential that the interviewers were relatable to the participants. This was done through the interviewers having similar geographical backgrounds to that of the participants.

## Conclusion

There is a need to reduce the shortage of neurosurgical equipment in LMICs if neurosurgical care is to improve. However, it is unclear if neurosurgical equipment donations provide the best path to meeting these needs, although they have provided a solution to this challenge historically. Other solutions that are not linked to dependency need to be explored and executed. A key factor behind neurosurgical equipment donations that meet the needs of the recipient and the provision of adequate training is the development of collaborative relationships between LMICs and HICs. These relationships may be best created within an organisation framework that has the logistical capabilities of coordinating international equipment donation and providing a standardised, quality control measure.

## Data Availability Statement

The original contributions presented in the study are included in the article/Supplementary Material, further inquiries can be directed to the corresponding author/s.

## Ethics Statement

The studies involving human participants were reviewed and approved by University of Oxford Medical Sciences Inter-Divisional Research Ethics Committee. The patients/participants provided their written informed consent to participate in this study.

## Author Contributions

DS: conception, design, interviewing, data analysis, writing, reviewing and editing, visual abstract, and project administration. SB: conception, interviewing, data analysis, writing, reviewing and editing, supervision, and project administration. AC, JE, and JK: interviewing, data analysis, writing, reviewing and editing. AE: data analysis, writing, reviewing and editing. NB, GH, and DD: writing, reviewing and editing. UK: writing, reviewing and editing, and supervision. All authors contributed to the article and approved the submitted version.

## Conflict of Interest

The authors declare that the research was conducted in the absence of any commercial or financial relationships that could be construed as a potential conflict of interest.

## Publisher's Note

All claims expressed in this article are solely those of the authors and do not necessarily represent those of their affiliated organizations, or those of the publisher, the editors and the reviewers. Any product that may be evaluated in this article, or claim that may be made by its manufacturer, is not guaranteed or endorsed by the publisher.
