# Supplementary material for: Neurosurgical Equipment Donations: A Qualitative Study
Source: Front Surg. 2022 Jan 20;8:690910. doi: 10.3389/fsurg.2021.690910 (PMC8810520; doi:10.3389/fsurg.2021.690910)
Supplement: Supplementary file 1 [file Table_1.DOCX]

**Appendix S1**

**Questions for LMICs Institutions**

- What has been your experience of equipment donations within neurosurgery?
- How was your practice prior to equipment donations?
- How did the donor/recipient relationship begin/develop?
- Was the equipment donation based on the specific needs of your institution?
  - Who identified the clinical need?
- If required, how was relevant training organised?
- How did practice change following the donation?
  - Number/breadth/quality of operations?
- Have there been any instances where equipment donations have been rejected? And if so, why?
- Did you encounter any issues with the donated equipment?
  - Shipping?
  - Installation?
  - Regular use?
  - Maintenance/repair? - how is the equipment maintained?

**Questions for HICs institutions**

- What has been your experience of equipment donations within neurosurgery?
- How did the donor/recipient relationship begin/develop?
  - What motivated the donation?
- Where did the equipment come from? (e.g. new/used)
- How did you decide which equipment to donate? (i.e based on specific clinical need in recipient country)

- Have there been any instances where equipment donations have been rejected/returned? And if so, why?
- Do you have ways to identify potential recipients?
- Have you experienced any barriers to donating equipment? (i.e. funding, shipping)

**Appendix S2**

**1. Inequality in access to neurosurgical equipment**

*The limitations that we have in terms of offering neurosurgical services is due to lack of neurosurgical equipment. And this can be met.*

*Participant A (Zimbabwe)*

*In my hospital, there is no microscope. We tie a loupe to our head with light on it to operate. My hospital is the biggest hospital in the town, and it is a public hospital. And we have many, many, many patients coming in for neurosurgery.*

*Participant B (Cameroon)*

*The equipment should be donated.*

*Participant B (Cameroon)*

*Donations create a superiority and an inferiority relation, and a dependence situation, where you're always waiting on things to be donated to you; you're more like a beggar. An exchange setup is better. This can be at an individual or personal level or it can also be magnified to a government exchange programme.*

*Participant A (Zimbabwe)*

**2. Identifying specific neurosurgical equipment needs**

*South Africa is going to be very different to Zimbabwe.*

*Participant C (UK)*

*There's no point in providing that if in fact, that isn't the problem. And the problem is actually there's no CT scanners. So they are operating without knowing what the diagnosis is. So I would have thought, efforts to raise funds or equipment would need to target what is required by the population.*

*Participant D (UK)*

*I think the biggest challenge is going to be not knowing who needs what.*

*Participant E (UK)*

*I think materials like microscope, bipolar instruments, and consumables like bone wax or Surgicel are difficult to have here, and it would be helpful for us to have them donated.*

*Participant B (Cameroon)*

*They've got everything. Endoscopes are there.*

*Participant E (UK)*

*The priority for equipment donation for us is equipment for tumour and spine disease. Specifically, microscopes and drains.*

*Participant B (Cameroon)*

*One of the most important things we need here is the microscope.*

*Participant F (Benin)*

*It may be harder to donate microscopes and things like that that are very high quality. But we're moving to more advanced versions, and old ones after some labour are still very viable. But then there's all the transport costs which make it prohibitive.*

*Participant C (UK)*

*This equipment was donated by the North American hospitals. So they don't have a warranty and they don't have a service plan. So if something goes wrong, sorry. So we have to find a way to fix it. And if we can't repair it, we simply abandon it, and then go for the next one.*

*Participant E (UK)*

**3. Importance of organisations**

*I don't think me as an individual can do anything about that and therefore it's up to big organisations, big charities who would have the logistics and the financial backing to help individuals like me who could do something beyond a small scale.*

*Participant G (Ireland)*

*A clear argument from a logistics supply and delivery point of view, that doing this on a larger scale, through a more organised system will be more efficient in a business model.*

*Participant H (UK)*

*They have an operation there called PAHO - which is the Pan American Health Organisation -, which is part of the WHO, and PAHO actually collects this equipment and makes some safety checks. Make sure that is safe for the patients to use in these countries. Free from risk. Free from service problems and all those stuffs.*

*Participant E (UK)*

*My understanding of equipment donation is that WFNS provided some people with equipment for surgery, like microscopes and surgical instruments.*

*Participant F (Benin)*

*We are comfortable to partner with any centre of the WFNS.*

*Participant B (Cameroon)*

*When the WFNS gives material, we don’t know after 5 years or after 10 years, what the material becomes. Knowing what happens to the material is a good thing. Because when you check on your donation every year, you see that good guy, I gave him a microscope, and because of me he can do a lot of surgery, more than if he didn’t get the microscope. So it made a difference. So when you check every year, you see the plus you give this to a person.*

*Participant F (Benin)*

*I think that we, we have to, to create more organisation to be more efficient, because to have that, just two organisation for the whole world is very few.*

*Participant F (Benin)*

**4. Partnerships between LMIC and HIC centres**

*I once went to visit colleagues in America. Whilst there, I gave them tea leaves from my home in Zimbabwe and these tea leaves have been shown to have some therapeutic effects. It did not cost much, but they really appreciated it, and thought that was a good gift. When I got back home, I was offered some neurosurgical equipment as a gift. This is an example of how exchange could work.*

*Participant A (Zimbabwe)*

*You're invested in somebody. You know, it's your friend out there. You want to keep going rather than just doing it once.*

*Participant H (UK)*

*I think that it's better to use a partnership because when we have a partnership we can share together. When there is a partnership, there is more participation from both sides and the donations will be tailored to our needs.*

*Participant B (Cameroon)*

*I think that we have to create something formal. Because relying on friends of friends is not very good.*

*Participant F (Benin)*

*I think we have a lot of people who want to help neurosurgeons in low income countries, but they don't know how to do it. So an organisation should facilitate between people who want to donate and the people who can be a beneficiary of it.*

*Participant F (Benin)*

**5. Equipment donations are insufficient in isolation**

*If you were to then look at it from a neuro endoscope point of view, let's do something very specific for me. It's not only having your endoscope it's knowing how to use it. So that is where the individual relationships come in. Because it is about talking to somebody in another unit, having a conversation about patients. So that the whole point of equipment donation is to be able to treat patients more effectively or treat more patients. And effectively, it really needs teaching and education about how to use this new piece of care, not just giving it and saying, here you go is a shiny piece of kit. So having those personal relationships allows you to train the centre that's accepting the kits into the best use, and therefore allowing them to get the best out of that equipment.*

*Participant H (UK)*

*The most important thing is not donating the kit, it's making sure that it can be used.*

*Participant D (UK)*

*Education and training would have a much better and bigger impact in the long term.*

*Participant D (UK)*

*The people who want to help have to come to our place to see our condition, our work conditions, to see what we need to perform our surgery here. And maybe like, come two or three times to perform with us to see what we work, how we work, and to see what we have to change to improve it.*

*Participant F (Benin)*

*If the trainer goes to the trainee unit and teaches you how to use it, the advantage of that is, of course, that you’re using the equipment that they will then continue to use. There's not like, Oh, well, you know, this now looks different from the one I tried. This is the actual kit you'll be using. And also, the trainer gets to teach in a sense that recognises the limitations of the trainee unit and say, ah, you don't have a, b, and c, so let's try using this. Normally we would do this, but you don't have that, right we'll do this. So it enables the trainer to use all of their experience, and bastardise what may be the usual method to fit what the trainee unit can do. That’s the advantage of going over.*

*Participant H (UK)*

*You'd have to leave a legacy after you'd have to make sure that who's going to look after those kids post-operatively, so I thought that you know if you're going to do it right, do it right.*

*Participant G (Ireland)*

*It requires the trainer to go out to the trainee unit. And that sounds wrong*

*Participant H (UK)*
